# Whole-Gene Deletions of *FZD4* Cause Familial Exudative Vitreoretinopathy

**DOI:** 10.3390/genes12070980

**Published:** 2021-06-27

**Authors:** Li Huang, Jinglin Lu, Linyan Zhang, Zhaotian Zhang, Limei Sun, Songshan Li, Ting Zhang, Limei Chen, Liming Cao, Xiaoyan Ding

**Affiliations:** Xiaoyan Ding, State Key Laboratory of Ophthalmology, Zhongshan Ophthalmic Center, Sun Yat-sen University, 54 Xianlie Road, Guangzhou 510060, China; Huangli@gzzoc.com (L.H.); lujlin5@mail2.sysu.edu.cn (J.L.); zhangly226@mail2.sysu.edu.cn (L.Z.); Zhangzhaotian@gzzoc.com (Z.Z.); sunlimei@gzzoc.com (L.S.); Lisongshan@gzzoc.com (S.L.); zhangting@gzzoc.com (T.Z.); chenlimei@gzzoc.com (L.C.); caoliming@gzzoc.com (L.C.)

**Keywords:** familial exudative vitreoretinopathy, copy number variation, *FZD4*, whole-gene deletion, retinal fold

## Abstract

Familial exudative vitreoretinopathy (FEVR) is an inherited disorder characterized by abnormalities in the retinal vasculature. The *FZD4* gene is associated with FEVR, but the prevalence and impact of *FZD4* copy number variation (CNV) on FEVR patients are unknown. The aim of this study was to better understand the genetic features and clinical manifestations of patients with *FZD4* CNVs. A total of 651 FEVR families were recruited. Families negative for mutations in FEVR-associated genes were selected for CNV analysis using SeqCNV. Semiquantitative multiplex polymerase chain reaction and multiplex ligation-dependent probe amplification were conducted to verify the CNVs. Four probands were found to carry whole-gene deletions of *FZD4*, accounting for 5% (4/80) of probands with *FZD4* mutations and 0.6% (4/651) of all FEVR probands. The four probands exhibited similar phenotypes of unilateral retinal folds. FEVR in probands with CNVs was not more severe than in probands with *FZD4* missense mutations (*p* = 1.000). Although this is the first report of *FZD4* CNVs and the associated phenotypes, the interpretation of *FZD4* CNVs should be emphasized when analyzing the next-generation sequencing data of FEVR patients because of their high prevalence.

## 1. Introduction

Familial exudative vitreoretinopathy (FEVR; MIM: 133780) is an inherited disorder characterized by abnormalities of the retinal vasculature [1]. Its hallmark characteristics are avascularity in the peripheral retina [2], retinal folds, vitreous hemorrhage, and retinal detachment. Its clinical manifestation spectrum ranges from mild, with normal visual acuity, to severe, with blindness since infancy [3]. The inheritance patterns of FEVR are autosomal dominant (AD), autosomal recessive (AR), and X-linked recessive (XL). Mutations in nine genes, norrin cystine knot growth factor NDP (*NDP*; MIM: 300658) [4], frizzled class receptor 4 (*FZD4*; MIM: 604579) [5], low-density lipoprotein receptor-related protein 5 (*LRP5*; MIM: 603506) [6], tetraspanin 12 (*TSPAN12*; MIM: 613138) [7], zinc finger protein 408 (*ZNF408*; MIM: 616454) [8], kinesin family member 11 (*KIF11*; MIM:148760) [9], catenin, β-1 (*CTNNB1*; MIM: 116806) [10], jagged 1 (*JAG1*; MIN: 601920) [11], and catenin, α-1 (*CTNNA1*; MIM:116805) [12], have been reported to cause FEVR. *NDP* is inherited as an XL trait, *LRP5* can be inherited as either AD or AR, and the remaining genes are passed on as AD. Mutations in six of the nine genes (*FZD4*, *TSPAN12*, *NDP*, *LRP5*, *CTNNB1*, and *CTNNA1*), which are related to the Norrin/Wnt signaling pathway and play essential roles in retinal development and angiogenesis [13], can explain 50% of FEVR cases [14].

*FZD4*, a member of the frizzled gene family located on human chromosome 11q14.2, was first isolated from a human gastric cancer cDNA pool in 1999 [15]. It contains two exons and encodes a 537-amino acid protein with a seven-transmembrane domain. Acting as a receptor in the Norrin/Wnt signaling pathway, *FZD4* is involved in cell signal transduction, cell proliferation, and cell death and is essential for the development of the retinal vascular system [16]. Salvo et al. reported that *FZD4* mutations accounted for 15.2% of FEVR patients [14], while in our previous study, *FZD4* mutations were detected in 21% of FEVR families [17]. According to the Human Gene Mutation Database (HGMD; http://www.hgmd.cf.ac.uk assessed on 17 May 2021), the mutation types of *FZD4* include nonsense mutation, missense mutation, small deletion, small insertion, gross deletion, and complex rearrangement.

Copy number variation (CNV), a common type of pivotal genetic variation, results in an abnormal number of copies of large genomic regions. It includes deletion, insertion, and duplication of DNA fragments that are usually longer than 1 Kb [18]. CNV not only contributes to the variations observed between human beings but is also known to be the cause of numerous diseases [19]. CNV has been reported in FEVR-related genes, including *TSPAN12* [20], *KIF11* [21], *LRP5* [22], and *NDP* [23]. However, no CNV has been reported in *FZD4*. This may be partly due to the fact that CNV detection remains challenging [24]. Additionally, the clinical features of patients with *FZD4* CNVs remain unknown. Therefore, the purpose of this study was to investigate the effect of *FZD4* CNVs in FEVR cases, describe their exact clinical manifestations, and analyze the genotype–phenotype correlation.

## 2. Materials and Methods

### 2.1. Patients

This cross-sectional study was approved by the Institutional Review Board of Zhongshan Ophthalmic Center, Sun Yat-sen University (2014MEKY048) and adhered to the tenets of the Declaration of Helsinki. Written informed consent was obtained from all adult participants and the parents or guardians of children.

A total of 651 FEVR probands were recruited from patients referring to our hospital from January 2014 to April 2021. The clinical diagnostic criteria of FEVR were based on previous reports [25,26], and disease staging was performed according to the classification system of Trese et al. [27]. To eliminate the possibility of the presence of retinopathy of prematurity, patients with a gestational age of less than 32 weeks or with a birth weight of less than 1500 g were excluded.

### 2.2. Clinical Assessments

Complete ophthalmic examinations were performed on all probands and their family members, including best-corrected visual acuity (BCVA), intraocular pressure (IOP), slit-lamp biomicroscopy, and binocular indirect or direct ophthalmoscopy. Children underwent either scanning laser ophthalmoscopy (SLO; Nidek F-10; Nidek, Gamagori, Japan) or RetCam examination (Clarity Medical Systems, Pleasanton, CA, USA). Adults underwent fundus photography with an FF450 fundus camera (Zeiss, Oberkochen, Germany). Optical coherence tomography (OCT) was performed with a Spectralis HRA (Heidelberg Engineering, Heidelberg, Germany), and B-scan ultrasonography was conducted using Compact Touch 4.0 (Quantel Medical, Cournon-d’Auvergne, France). Most patients underwent fundus fluorescein angiography (FFA) with RetCam (Clarity Medical Systems, Pleasanton, CA, USA) or Spectralis HRA (Heidelberg Engineering, Heidelberg, Germany).

### 2.3. DNA Extraction

Peripheral whole blood samples were collected from all participants for DNA extraction and genetic analysis [17]. Whole-exome sequencing (WES) was performed in the probands, and Sanger sequencing was used for validation and segregation analysis. The analysis process of WES data was described in our previous study [28]. CNVs were analyzed using the SeqCNV method based on the WES data [29].

### 2.4. CNV Assessments

For CNV validation, semiquantitative multiplex polymerase chain reaction (qPCR) was performed on the *FZD4* gene with primers specific to the presumed deletion. Primers of exons 1 and 2 were designed. Exon 6 of *SPATA7* and exon 14 of *TTLL5* were used as positive controls. All primers are shown in Supplementary Table S1. For each PCR reaction, 5 ng of DNA, 0.3 ul of 2-μmol/L forward/reverse primers, 5.9 μL of water, and 7.5 μL of SYBR Green PCR Master Mix (Thermo Fisher Scientific, Waltham, MA, USA) were used. A standard thermocycling program was used for amplification with a PCR system (StepOnePlus Real-Time PCR System, Thermo Fisher Scientific, Waltham, MA, USA). The qPCR amplification of the target and control gene regions was performed between the test and control samples. The 2 × 2^−ΔΔCt^ equation, where ΔΔCT = (Ct reference − Ct target) sample − (Ct reference − Ct target) calibrator, was used to calculate the relative amount of each sample. When the relative amount of the test sample was half or an integral multiple of that of the control group, the CNVs of *FZD4* were identified. Multiplex ligation-dependent probe amplification (MLPA, Supplementary Table S2) was used to determine the copy numbers of up to 40 DNA sequences using a single multiplex PCR-based reaction [30]. An MLPA kit (SALSA MLPA Probemix P285-C3 LRP5; FALCO Biosystems, MRC Holland, Amsterdam, Netherlands) was used in accordance with the manufacturer’s instructions. The reaction products were separated and visualized on a genetic analyzer (ABI PRISM 3130 Genetic Analyzer, Applied Biosystems, Foster city, CA, USA), followed by analysis using Coffalyser.net software (MRC Holland, Amsterdam, The Netherlands).

All statistical analyses were performed using IBM SPSS Statistics version 25.0 (IBM Corporation, Armonk, NY, USA). The disease stages were analyzed using Fisher’s exact test. A value of *p* < 0.05 was considered statistically significant.

## 3. Results

### 3.1. Clinical Features of Probands with FZD4 CNVs

*FZD4* mutations were detected in 80 of the 651 FEVR families, and *FZD4* CNVs were identified in four families (probands XDW248, DX1344, DX1561, and DX1988), accounting for 5% of the patients with *FZD4* mutations and 0.6% of the entire FEVR cohort. The patients’ clinical data are summarized in Table 1.

**Figure 1:**
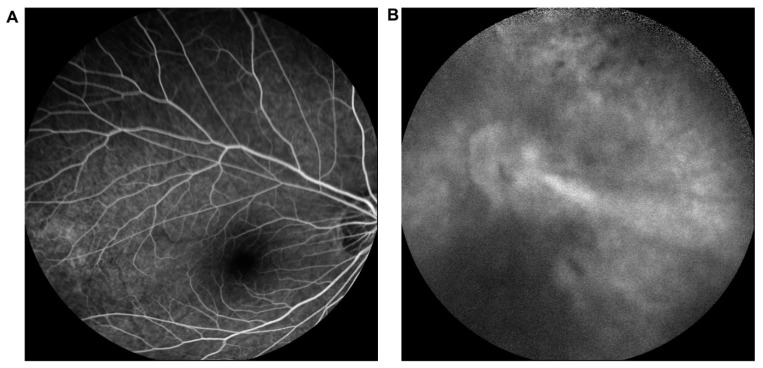
Fundus fluorescein angiography (FFA) of XDW248. FFA showed macular dragging and supernumerary branching in the right eye (**A**) and vitreous opacity and a retinal fold in the left eye (**B**).

XDW248 was referred to our hospital because of decreased visual acuity in both eyes at the age of 12. Her BCVA was 20/32 in the right eye, and she had no light perception in the left eye. Both her corneas were clear, while the lenses were opaque. A retinal fold extending from the optic disc to the temporal retina of her left eye was vaguely observed on fundus photography and FFA. FFA revealed increased vessel branching in the posterior pole and vascular leakage of the peripheral retina of her right eye (Figure 1). A visual field test revealed defects in the right eye, and OCT showed abnormal retinal nerve fiber layer thickness in both eyes. Rhegmatogenous retinal detachment developed in her right eye two years later, and she underwent scleral buckling.

**Figure 2:**
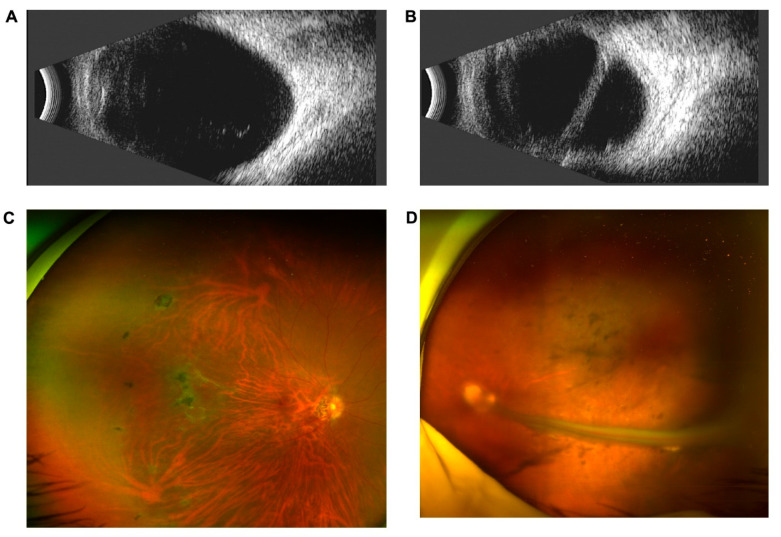
Clinical features of DX1344. B-scan ultrasonography showed vitreous opacity in the right eye (**A**) and retinal folds in the left eye (**B**). Scanning laser ophthalmoscopy showed pigmentation on the nasal retina of the right eye (**C**) and retinal folds in the left eye (**D**).

Proband DX1344 was the only adult among the four probands with CNVs. He had been diagnosed with amblyopia of the left eye since childhood and had sought medical attention due to redness and pain in that eye at the age of 35 years. The BCVA of the right eye was 20/100, while the left eye has no light perception. His IOP was 10.5 mmHg in the right eye and 39.7 mmHg in the left. He was diagnosed with secondary glaucoma in the left eye. Retinal folds in the left eye were clearly visible on trough B-mode ultrasonography and fundus photography (Figure 2).

**Figure 3:**
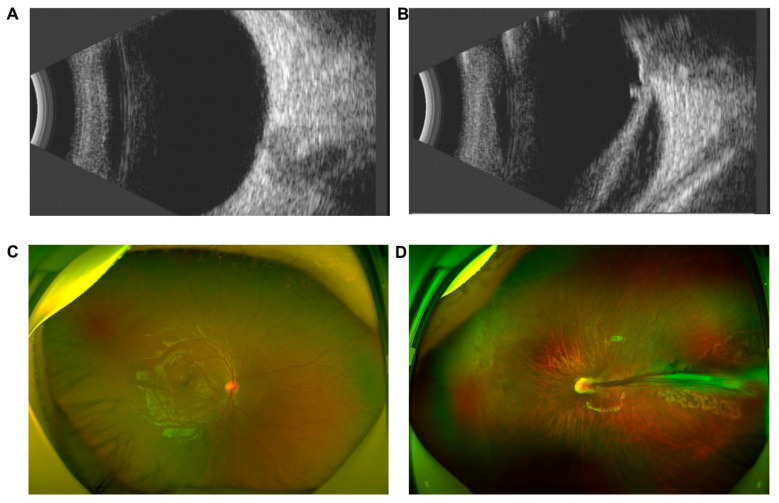
Clinical features of DX1561. B-scan ultrasonography showed a normal retina and vitreous in the right eye (**A**) and retinal folds in the left eye (**B**). Scanning laser ophthalmoscopy showed a normal retinal anatomy of the right eye (**C**) and retinal folds in the left eye (**D**).

Proband DX1561 was diagnosed with exotropia of the left eye at the age of 3 years and referred to our hospital one year later. BCVA examination was not performed because the patient was uncooperative. Retinal folds were observed in her left eye (Figure 3).

**Figure 4:**
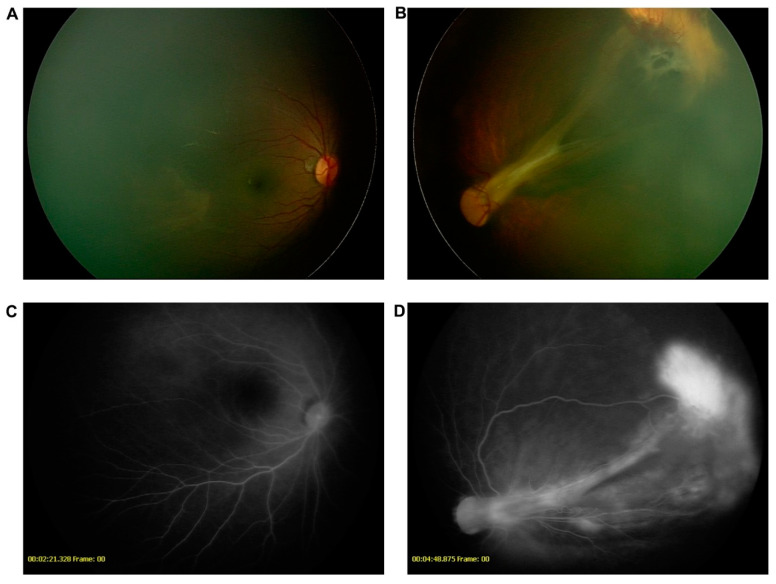
Clinical features of DX1988. RetCam showed avascular area in the temporal part in the right eye (**A**) and retinal folds in the left eye (**B**). Fundus fluorescein angiography showed supernumerary branching in the right eye (**C**) and retinal folds in the left eye with leakage (**D**).

DX1988 was referred to our hospital with a diagnosis of exotropia of the left eye at the age of 6 months. He had transparent corneas and cloudy lenses in both eyes. B-mode ultrasonography showed retinal detachment in his left eye. Flash-visual-evoked potentials showed a moderate to severe decrease in the P2 wave amplitude in his left eye. FFA demonstrated retinal folds, a peripheral avascular zone, vascular leakage, and straightened vessel branching in his left eye, and increased straightened vessel branching and a peripheral avascular zone in his right eye (Figure 4). Intravitreous ranibizumab to treat the extraretinal neovascularization of the left eye was conducted at one year old.

A clear pattern emerged from the clinical manifestations of all four probands: they all had retinal folds in the left eye and mild clinical manifestations in the right eye. To assess the phenotype severity of the four probands with *FZD4* CNVs, we compared their FEVR stages with those of probands with *FZD4 missense* mutations. The most advanced stage in the four probands was stage 4. The difference in FEVR stage between the four probands with *FZD4* CNVs and all other probands with *FZD4* missense mutations was that CNVs were not more severe than those of probands with missense mutations (Table 2). 

### 3.2. FZD4 CNVs

**Figure 5:**
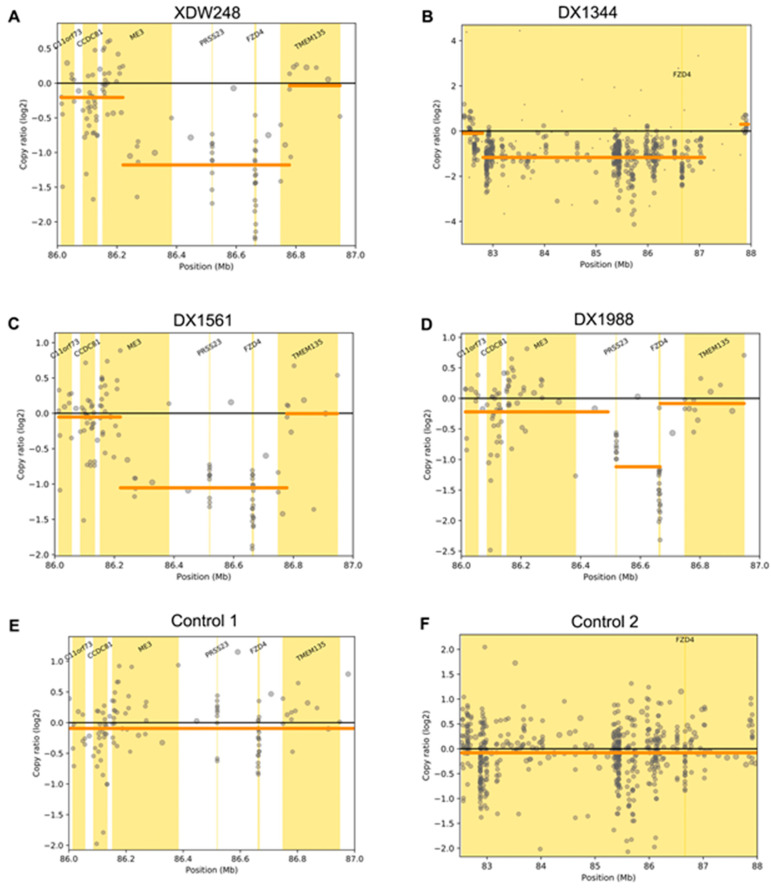
*FZD4* CNVs detected by SeqCNV. (**A**) A 0.51 Mb deletion was detected in XDW248. (**B**) A 4.5 Mb deletion was detected in DX1344. (**C**) A 0.51 Mb deletion was detected in DX1561. (**D**) A 0.15 Mb deletion was detected in DX1988. (**E**) Normal control without a deletion within the same region as (**A**,**C**,**D**). (**F**) Normal control without a deletion within the same region as (**B**).

**Figure 6:**
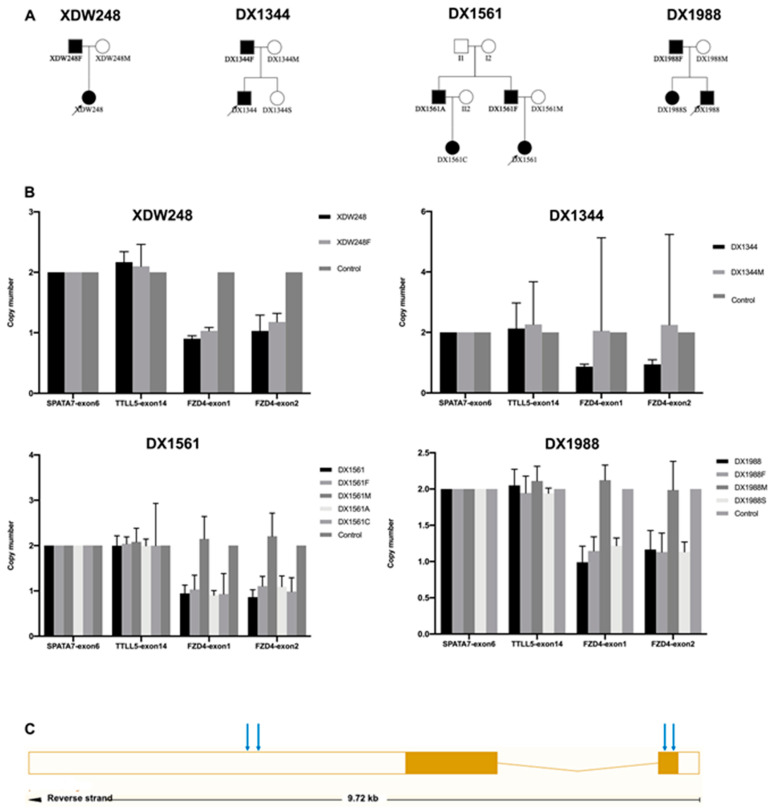
Confirmation of whole-gene deletions of *FZD4* using semiquantitative multiplex *PCR*. (**A**) The pedigrees of the four families with *FZD4* CNVs. (**B**) Representative histograms of *FZD4* exons 1 and 2 compared to other family members. *TTLL5* exon 14 and *SPATA7* exon 6 were used as controls. XDW248 and XDW248F were affected in family XDW248. DX1344 was affected in family DX1344. DX1561, DX1561F, DX1561A, and DX1561C were affected, and DX1561M was unaffected in family DX1561. DX1988, DX1988F, and DX1988S were affected, and DX1988M was unaffected in family DX1988. (**C**) The structure of *FZD4* which contains two exons. The blue arrow indicated where the PCR primers located.

**Figure 7:**
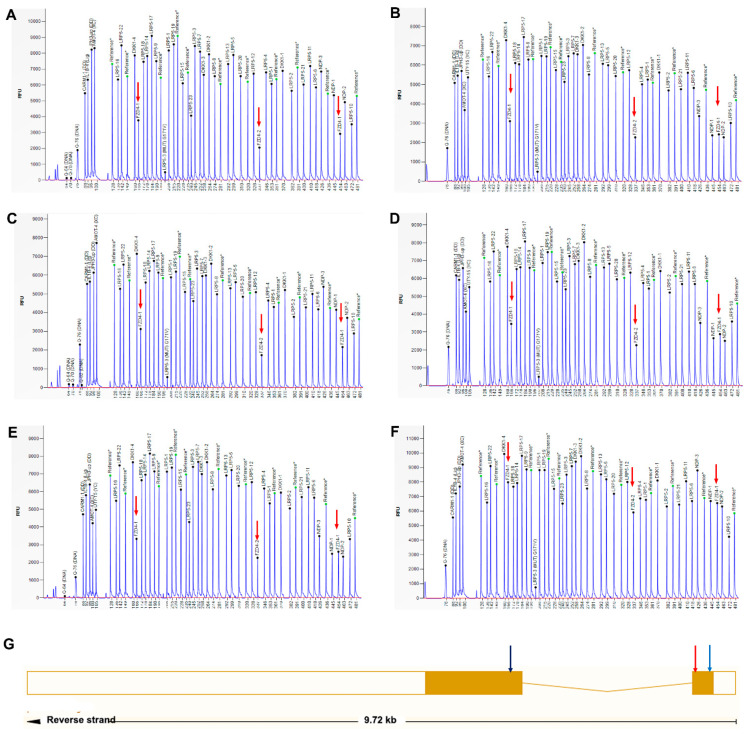
Confirmation of whole-gene deletions of *FZD4* using MLPA. The MLPA analysis of XDW248 (**A**), DX1344 (**B**), DX1561 (**C**), DX1988 (**D**), and XDW248F (**E**). The arrows indicate a half-reduced relative peak height corresponding to *FZD4* gene exon 1 and exon 2 probes. E. DX1344M is normal with no signal alterations in any *FZD4* probes. *NDP is a gene on the X chromosome; the males (**B**,**D**,**E**) had half-reduced relative peak in *NDP* probes, which is normal compared to the females (**A**,**C**,**F**). (**G**) The MLPA probes ligation sites were listed. Probe 1 (blue arrow) and probe 2 (red arrow) were ligated to *FZD4* exon 1. Probe 3 (black arrow) was ligated to *FZD4* exon 2.

WES identified no genetic mutations, while SeqCNV detected potential deletions in chromosome 11 of the four probands (Figure 5). Whole-gene deletion of *FZD4* was confirmed by qPCR analysis. The relative quantity of the *FZD4* exon 1 and 2 amplicon copy number ratios in the four probands was close to 1 (the normal copy number ratio should be 2), confirming the deletion of *FZD4* in the heterozygous state. The copy number ratios of *TTLL5* exon 14 and *SPATA7* exon 6 were around 2 (Figure 6). The MLPA confirmed the heterozygous deletion state of *FZD4* gene in the four probands (Figure 7).

## 4. Discussion

In this study, we identified four families with whole-gene deletions of *FZD4*. To our knowledge, no whole-gene deletions of *FZD4* have been reported previously. A total of 128 *FZD4* mutations have been reported (HGMD), including 76 (59.3%) missense mutations, 15 (11.7%) nonsense mutations, 22 (17.2%) small deletions, 11 (8.6%) small insertions, 3 (2.3%) gross deletions, and 1 (0.8%) complex rearrangement. This is the first study to report FEVR patients with genotypically confirmed pathogenic whole-gene deletions.

All probands presented with similar phenotypes, with retinal folds in the left eye and a demarcation line in the right eye, indicating the border between the vascular and avascular zones. The *FZD4*-associated phenotype exhibits varying severity between different mutations and asymmetry between contralateral eyes [31]. In this study, similar phenotypes and moderate asymmetry between contralateral eyes were observed. Retinal folds were the typical phenotype. In our previous study, 73.7% (14/19) of patients with *FZD4* mutations had unilateral folds [32]. In addition to the same genetic mutations, other factors affecting the phenotypes should be further evaluated. In our study, the phenotypes of patients with whole-gene deletions were no more severe than those of patients with other types of mutation. Similar findings have been reported for other FEVR-related genes. Seo et al. [20]. and Li et al. [31]. found that the FEVR severity of patients with large deletions of *TSPAN12* was not greater than that of patients with point mutations. Likewise, the clinical features associated with *KIF11* were not more severe in patients with large deletions than in patients with point mutations [31].

The detection rate of causative mutations in the known FEVR genes should be higher than in previous studies because of the CNVs. Thus far, nine gene mutations have been reported to cause FEVR. However, these genes are known to be responsible for less than half of FEVR patients [14]. The reported CNVs, including large deletions, large insertions, and whole-gene deletions, account for a small proportion of all FEVR-related gene mutations: 1.4% (4/283) in *LRP5*, 13.5% (25/185) in *NDP*, 7.6% (7/92) in *TSPAN12*, 6.3% (6/95) in *KIF11*, and 5.9% (4/68) in *CTNNB1* (HGMD). Moreover, no such mutations have been reported in *FZD4*, *ZNF408*, *JAG1*, and *CTNNA1*. Ellingford et al. found that at least 7% of individuals with inherited retinal diseases have a CNV within genes related to their clinical diagnosis [33]. Therefore, the incorporation of CNV analysis into FEVR screening is essential. First-generation (Sanger) sequencing and next-generation sequencing (NGS) could characterize single nucleotide variations and small insertions and deletions [34] but often failed to capture the complete spectrum of genomic variations [33]. Ellingford et al. used ExomeDepth for the detection of CNVs from gene panel NGS data sets, specific informatics filtering strategies, and MLPA for the confirmation of identified CNVs [33]. Chen et al. used SeqCNV to reliably identify CNVs of different sizes using capture NGS data [29]. Seo et al. used semiquantitative multiplex PCR and droplet digital PCR to identify *TSPAN12* CNVs and detected three large deletions in patients who had been previously screened and found negative for *NDP*, *FZD4*, *LRP5*, and *TSPAN12* mutations [20]. In this study, SeqCNV was used to detect CNVs from WES data, followed by qPCR for verification, resulting in the confirmation of four whole-gene deletions of *FZD4*. Therefore, evaluating CNVs of FEVR-related genes should be considered in FEVR screening and diagnosis as well as in routine genetic workups of patients.

There are genes, *ME3*, *PRSS23*, and *TMEM135*, deleted with *FZD4*. *ME3*, a malic enzyme, is expressed predominantly in organs with a low division rate [35]. *PRSS23* is a serine protease reported to be a positive regulator of endothelial-to-mesenchymal transition [36]. *TMEM135* is expressed by human multipotent adipose tissue-derived stem cells which may play role in adipogenic or osteoblastogenic differentiation [37]. None of these genes were reported to associate with any specific phenotype.

## 5. Conclusions

In summary, we identified whole-gene deletions of *FZD4* in four families, accounting for 5% of the patients with *FZD4* mutations and 0.6% of the entire FEVR cohort. CNV-associated phenotypes were not more severe than other mutation types. The evaluation of CNVs of FEVR-related genes in clinically confirmed FEVR patients should be carefully interpreted because of their high prevalence.

## Figures and Tables

**Table 1:** Clinical Characteristics of Patients with *FZD4* whole-gene deletions.

Family ID		XDW248	DX1344	DX1561	DX1988
Sex		Female	Male	Female	Male
At diagnosis	Age (yrs.)	12	4	1	1
	Stage (OD/OS) †	1/4	1/4	1/4	1/4
	Retinal folds (OD/OS)	no/yes	no/yes	no/yes	no/yes
At last follow-up	Age (yrs.)	14	36	4	3
	Stage (OD/OS)	4/4	1/4	1/4	1/4
	Refractive Error (Spherical Equivalent, OD/OS)	−13.38; NLP §	NA	+0.25; −16.75	NA/NA
	BCVA ‡	0.2/NLP	0.2/NLP	NA/NA	NA/NA
	Others	OD retinal detachment	OS glaucoma	NO	NO
Inherited from		Paternal	Paternal	paternal	paternal
Stage of affected parent (OD/OS)		2/2	NA/NA	1/1	1/1

†, OD, right eye, OS, left eye. ‡, BCVA, best corrected visual acuity. §, NLP, no light perception. NA, not available.

**Table 2:** Clinical data of patients with CNVs and missense mutations.

Mutations	CNVs	Missense	*p*
Age (yrs. M ± SD)	5.00 ± 6.16	9.14 ± 12.50	0.857
Gender (Male: female)	2:2	22:8	0.564
Stage *			1.000
1	0	2	
2	0	4	
3	0	4	
4	4	17	
5	0	3	
Total patients	4	30	

yrs., Years old. * The highest stage of each patient’s eye was used.

## Data Availability

The data that support the findings of this study are available from the corresponding author upon reasonable request.

## Supplementary Materials

The following are available online at https://www.mdpi.com/article/10.3390/genes12070980/s1, Table S1: qPCR primers in this study.
